# Hypothesizing That Pediatric Autoimmune Neuropsychiatric Associated Streptococcal (PANDAS) Causes Rapid Onset of Reward Deficiency Syndrome (RDS) Behaviors and May Require Induction of “Dopamine Homeostasis”

**DOI:** 10.4236/oji.2022.123004

**Published:** 2022-09

**Authors:** Kenneth Blum, Catherine A. Dennen, Eric R. Braverman, Ashim Gupta, David Baron, Bernard William Downs, Debasis Bagchi, Panayotis Thanos, Maureen Pollock, Jag Khalsa, Igor Elman, Abdalla Bowirrat, Rajendra A. Badgaiyan

**Affiliations:** 1 Center for Mental Health and Sports, Psychiatry, Western University Health Sciences, Pomona, USA; 2 The Kenneth Blum Behavioral & Neurogenetic Institute, Austin, USA; 3 Department Psychiatry, Boonshoff School of Medicine, Wright University, Dayton, USA; 4 Institute of Psychology, ELTE Eötvös Loránd University, Budapest, Hungary; 5 Department of Psychiatry, School of Medicine, University of Vermont, Burlington, USA; 6 Division of Nutrigenomics, Victory Nutrition International Inc., Bonita Springs, USA; 7 Department of Molecular Biology, Adelson School of Medicine, Ariel University, Ariel, Israel; 8 Department of Family Medicine, Jefferson Health Northeast, Philadelphia, USA; 9 Future Biologics, Lawrenceville, USA; 10 Department of Pharmaceutical Sciences, Texas Southern University, Houston, USA; 11 Department of Psychology & Behavioral Neuropharmacology and Neuroimaging Laboratory on Addictions (BNNLA), Research Institute on Addictions, University at Buffalo, Buffalo, USA; 12 Department of Microbiology, Immunology, and Tropical Medicine, School of Medicine, George Washington University, Washington DC, USA; 13 Cambridge Health Alliance, Harvard Medical School, Cambridge, USA; 14 Department of Psychiatry, Ichan School of Medicine at Mount Sinai, New York, USA; 15 Department of Psychiatry, South Texas Veteran Health Care System, Audie L. Murphy Memorial VA Hospital, Long School of Medicine, University of Texas Health Science Center, San Antonio, USA

**Keywords:** PANDAS, CANS, Reward Deficiency Syndrome, Group A Beta-Hemolytic Streptococcal (GABHS), Pro-Dopamine Regulation, Dopamine Homeostasis, Molecular Mimicry, Lyme, Borrelia

## Abstract

Pediatric autoimmune neuropsychiatric disorders associated with group A streptococcal infections (PANDAS) is a concept that is used to characterize a subset of children with neuropsychiatric symptoms, tic disorders, or obsessive-compulsive disorder (OCD), whose symptoms are exacerbated by group A streptococcal (GAS) infection. PANDAS has been known to cause a sudden onset of reward deficiency syndrome (RDS). RDS includes multiple disorders that are characterized by dopaminergic signaling dysfunction in the brain reward cascade (BRC), which may result in addiction, depression, avoidant behaviors, anxiety, tic disorders, and/or OCD. According to research by Blum *et al*., the dopamine receptor D2 (DRD2) gene polymorphisms are important prevalent genetic determinants of RDS. The literature demonstrates that infections like Borrelia and Lyme, as well as other infections like group A beta-hemolytic streptococcal (GABHS), can cause an autoimmune reaction and associated antibodies target dopaminergic loci in the mesolimbic region of the brain, which interferes with brain function and potentially causes RDS-like symptoms/behaviors. The treatment of PANDAS remains controversial, especially since there have been limited efficacy studies to date. We propose an innovative potential treatment for PANDAS based on previous clinical trials using a pro-dopamine regulator known as KB220 variants. Our ongoing research suggests that achieving “dopamine homeostasis” by precision-guided DNA testing and pro-dopamine modulation could result in improved therapeutic outcomes.

## 1. Pediatric Autoimmune Neuropsychiatric Disorders Associated with Streptococcal Infections (PANDAS)

Pediatric autoimmune neuropsychiatric disorders associated with streptococcal infections (PANDAS) are a group of emergent and problematic pediatric disorders. This group tends to involve a small subset of children, particularly in preadolescence, who develop an unknown rapid onset of reward deficiency syndrome (RDS), which includes addiction, depression, avoidant behaviors, anxiety, tic disorders, and/or obsessive-compulsive disorder (OCD) [1] [2] [3]. In addition, PANDAS in children can also manifest as emotional lability, separation anxiety, night-time fears, worsening handwriting, and learning regression. It has also been widely established that the dopamine receptor D2 (DRD2) dysfunction causes aberrant substance-seeking behaviors, including tobacco, alcohol, drugs, and food, as well as other related behaviors (*i.e*., pathological gambling, attention deficit hyperactivity disorder, and Tourette’s syndrome, including tics) [4]-[14]. The dopaminergic system, specifically DRD2, has been implicated in brain reward mechanisms in over 25,290 articles as of September 15, 2022. Additionally, research by Blum *et al*. has demonstrated that the DRD2 gene polymorphisms are important prevalent genetic determinants of RDS [15].

Group A beta-hemolytic streptococcal (GABHS) infections are thought to be the root cause of RDS and a variety of aggressive and depressive behaviors [3]. Cumulative data strongly suggests that infections such as Borrelia and Lyme, as well as other GABHS infections, cause an autoimmune reaction and associated antibodies target dopaminergic loci in the mesolimbic region of the brain, which disrupts brain function. Our group asserted in a previously published article that the principal vector of Lyme disease in the United States (US) is Ixodes scapularis (deer or black-legged ticks), and patients infected with this disease can present with anxiety and depression [16]. This study also discovered transcript coding for two putative cytosolic sulfotransferases, indicating that these ticks identified phenolic monoamines as their substrates. More specifically, dopamine and octopamine-targeting sulfotransferase activity were discovered in later recombinant proteins. Furthermore, it was demonstrated that, within the Ixodid tick’s salivary glands, the activation of Ixosc Sult 1 and Sult 2 may result in the deactivation of the salivation signal through the sulfonation of dopamine or octopamine. RDS behaviors can be caused by this phenomenon alone.

The original concept of PANDAS can be traced back to a clinical trial that was conducted at the National Institutes of Health (NIH) in the US [17]. The nosology has recently changed from PANDAS to Childhood Acute Neuropsychiatric Syndrome (CANS) [18]. At the moment, the diagnosis of PANDAS along with the hypothesis that infections can cause symptoms in a subgroup of children is disputed, particularly the proposed mechanism [19] [20] [21] [22]. Singer offered a more comprehensive concept to illustrate PANDAS by lowering the threshold assigned to PANDAS [22]. He claimed that CANS had a variety of unknown factors, which may or may not include GABHS and possibly hypodopaminergia [23].

## 2. Pandas Mechanisms

It is currently believed that PANDAS is an autoimmune reaction that, in part, mimics rheumatic fever and differs from the etiological links associated with spectrum disorders (e.g., attention deficit hyperactivity disorder, Tourette’s, autism, etc.) or RDS. As a result, rheumatic fever can be thought of as an autoimmune disorder caused by streptococcal infections in which antibodies affect the brain and induce neuropsychiatric conditions. In their article on Tourette’s Syndrome and OCD, Lombroso & Scahill introduced the idea of “molecular mimicry” as a potential explanation for PANDAS [24]. According to their theory, brain proteins and antigens on the cell wall of streptococcal bacteria are similar. As such, the produced antibodies cause an immunological reaction that targets and damages brain tissues, resulting in aberrant motor movements known as Sydenham chorea [25]. Some believe that, similar to Sydenham chorea, the antibodies cross-react with neuronal brain tissue, interrupting the brain reward circuit and causing tics and/or neuropsychiatric impairments that characterize PANDAS [26]. However, there is a lack of research supporting or disputing this hypothesis. The strongest supporting evidence comes from a controlled study that involved 144 children, but prospective longitudinal studies have not produced conclusive results [19].

In short, the following body of existing literature shows scientific support for the molecular basis of PANDAS-related mechanisms. The primary research credited with comprehending the molecular mechanisms implicated in PANDAS is from Madeleine Cunningham and colleagues at the University of Oklahoma Health Sciences Center. It is significant from a historical perspective that the original group, which consisted of the first 50 cases reported by Swedo *et al*. in 1998 [1], exhibited characteristics that were similar to Sydenham chorea, which provided evidence that assisted in distinguishing this original group from previously published OCD and tic cases [27]-[35].

As previously mentioned, GABHS infections are linked to a variety of neuropsychiatric disorders. The main theory regarding this association postulates that a GABHS infection generates auto-antibodies that, through molecular mimicry, cross-react with neuronal determinants (dopamine) in the brain. Cunningham’s group demonstrated in rats the link between GABHS-induced antibodies and the development of behavioral impairments [36]. To achieve this, over a period of 21 days, researchers administered immunoglobulin G (IgG) isolated from the sera of GABHS-exposed rats directly into the striatum of naïve rats. The most significant finding was that IgG from GABHS-exposed rats reacted with numerous receptors in vitro, including 5HT-2A, 5HT-2C, D1, and D2 receptors. However, it was discovered that, in vivo, specific brain proteins, such as serotonin transporters, dopamine receptors, and other neuronal proteins, colocalized with IgG deposits in the striatum of infused rats. Furthermore, Cox *et al*. conducted a study involving 311 subjects (aged 4 – 27 years, 66% male) who self-reported neuropsychiatric symptoms and also had concurrent group A streptococcal infection status [37]. When compared to healthy controls, the results revealed a significant increase in serum IgG antibodies against human dopamine receptor D1 (DRD1) and lysoganglioside. This seminal work, involving humans, supports the results from the rodent study [36]. The authors concluded that the results from this study showed a strong association between streptococcal-associated tics and OCD, elevated serum levels of anti-DRD1 and anti-lysoganglioside antineuronal antibodies, and higher CaMKII activation in human neuronal cells.

Further support has also been provided by other researchers and studies. For example, Quagliariello *et al*. demonstrated that streptococcal infections alter the bacterial populations in the gut and have an impact on the pro-inflammatory status by choosing particular bacterial strains linked to gut inflammation and immune response activation [38]. In terms of Sydenham chorea, Kirvan *et al*., in 2006, noted the pathogenesis of Sydenham chorea following group A streptococcal infection [39]. In this setting, Kirvan *et al*. believed Sydenham chorea was caused by antibodies that developed as a result of the infection and permeated into the brain, particularly the basal ganglia. Additionally, this group provided evidence that presumed antibodies present in acute chorea interact with the exterior of the neuronal cells, stimulate calcium calmodulin-dependent protein ki nase II, and increase tyrosine hydroxylase and the subsequent release of dopamine, which may result in a movement disorder.

In contrast to these results, Morris-Berry *et al*. assessed single-point-in-time optical densities for three putative antibodies identified in Sydenham chorea using enzyme-linked immunosorbent assays [40]. They found no differences between children with PANDAS (n = 44) or Tourette syndrome (n = 40) and controls (n = 24) for the streptococcal group A carbohydrate antigen, N-acetyl-beta-d-glucosamine, DRD2, or tubulin. However, a recent study by Chain *et al*. has successfully identified acute illness in both Sydenham chorea and PANDAS [41]. In particular, IgG autoantibodies against four neuronal autoantigens (lysoganglioside G_M1_, DRD1, DRD2, and tubulin) were observed, utilizing enzyme-linked immunosorbent assays, in Sydenham chorea sera (N = 8), sera and/or cerebrospinal fluid (CSF) from two groups of PANDAS cases (first group N = 25 and second group N = 35), sera from Tourette’s syndrome (N = 18), OCD (N = 25), attention deficit hyperactivity disorder (N = 18), and healthy controls (N = 28). While the reasons for this discrepancy in research findings are unclear, we recommend that interested readers scrutinize balanced reviews on related subjects [42]-[48].

## 3. Treatment of Pandas

With limited efficacy studies to date, the treatment of PANDAS remains controversial. However, standard treatment options for PANDAS include cognitive behavioral therapy, conventional tic therapy, and medications to treat OCD [49]. In the face of the arduous debate, there are off-label studies that demonstrate moderate to insufficient success when using antibacterial therapies [50] [51]. In addition, a single research study of individuals with PANDAS found, with limited to no supporting evidence, that immunomodulatory therapy, such as intravenous immunoglobulin (IVIG) or plasma exchange, is effective in treating symptoms [19] [24]. The NIH and American Academy of Neurology guidelines concluded in 2011 that there was “inadequate data to determine the efficacy of plasmapheresis in the treatment of acute OCD and tic symptoms in the setting of PANDAS”. Furthermore, Sigra *et al*. came to the conclusion in 2018 that rigorously conducted research regarding treatments for PANDAS is scarce, and published studies have a significant risk of bias [52].

Genomic testing, such as the Genetic Addiction Risk Score (GARS), could also help improve clinical outcomes and decision-making in individuals with PANDAS. GARS is a test that includes a panel of ten reward gene risk alleles and is able to accurately predict vulnerability to pain, addiction, and other compulsive behaviors that are defined as RDS and prevalent in PANDAS patients [53]. In addition, a unique therapeutic precision intervention utilizing the pro-dopamine regulator KB220 and associated variants, which includes a formulation of enkephalinase inhibitors, catabolic inhibitors, precursor amino-acids, and dopamine-releasing neuro-nutrients, can be used to induce dopamine homeostasis in individuals that have a genetic predisposition to developing RDS [54]. These genetically based formulations are based on the GARS test results. Therefore, coupling GARS testing with precision match KB220 pro-dopamine regulation could be used as a frontline modality to treat PANDAS/CANS in hypodopaminergic individuals with polymorphic risk alleles such as DRD1, DRD2, ANKK1 (Taq1A), etc.

## 4. Conclusion

**Figure 1.**
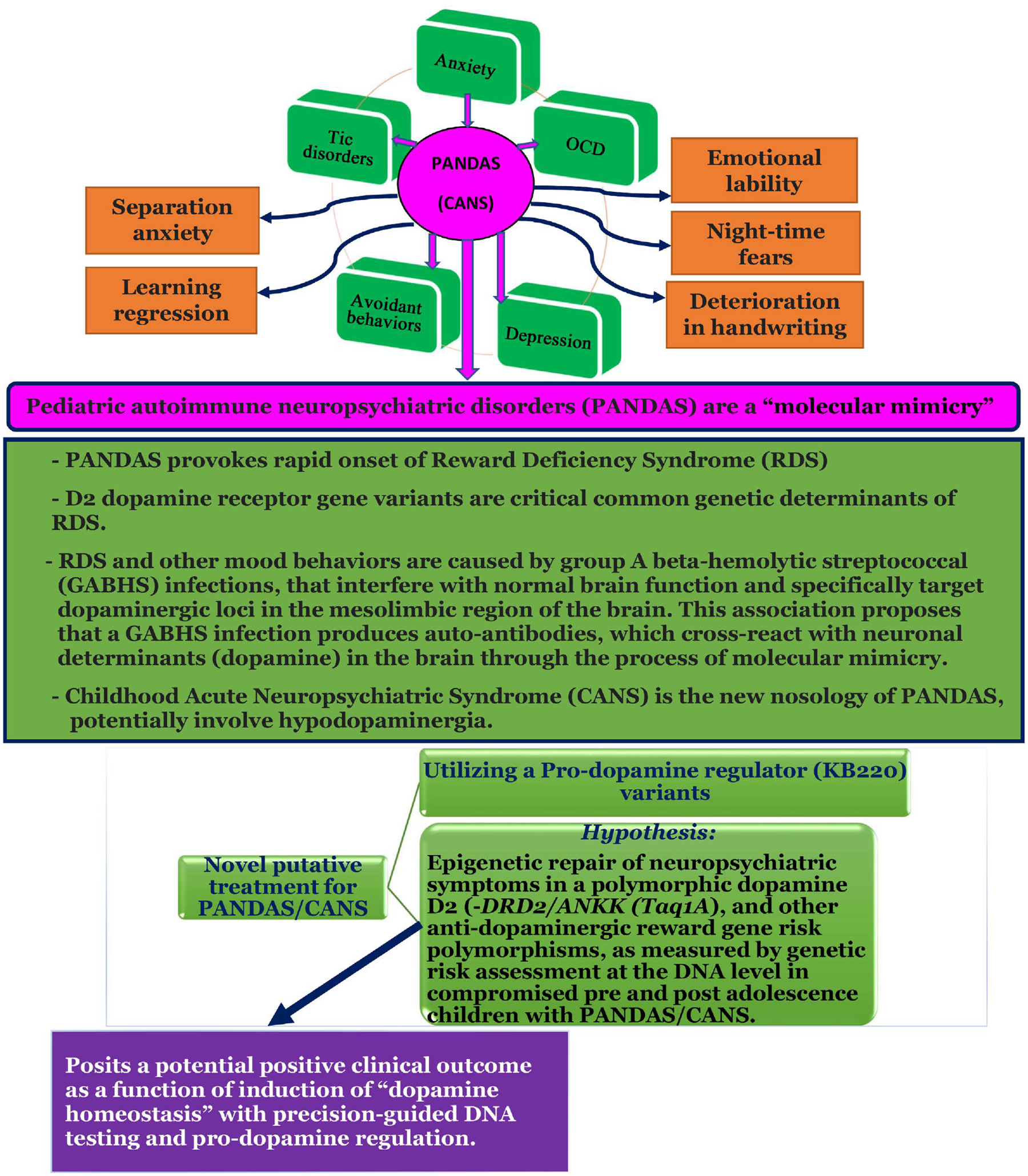
Hypothesizing that Pediatric Autoimmune Neuropsychiatric Associated Streptococcal (PANDAS) Causes Rapid Onset of (RDS) Behaviors and May Require Induction of “Dopamine Homeostasis”.

We propose a novel putative treatment for PANDAS/CANS in the future, based on previous clinical trials utilizing KB220 variants as pro-dopamine regulators. Our hypothesis relates to the epigenetic repair of neuropsychiatric symptoms in a polymorphic dopamine D2 [-DRD2/ANKK1 (Taq1A)] and other anti-dopaminergic reward gene risk polymorphisms as measured by genetic risk assessment at the DNA level in compromised pre- and post-adolescence children with PANDAS/CANS. Our ongoing work posits a potential positive clinical outcome as a function of the induction of “dopamine homeostasis” with precision-guided DNA testing and pro-dopamine regulation (Figure 1).
